# Non-Opioid Pharmaceutical Alternatives for Acute Pain Management in the Emergency Department: A Scoping Review

**DOI:** 10.5811/westjem.47925

**Published:** 2026-05-14

**Authors:** Akash Shanmugam, Sally M. Graglia, Curtis Geier, Juan Carlos C. Montoy, Alan M. Gelb, Kathy T. LeSaint

**Affiliations:** * University of California, San Francisco School of Medicine, San Francisco, California; † University of California, San Francisco School of Medicine, Department of Emergency Medicine, San Francisco, California; ‡ University of California, San Francisco, School of Pharmacy, San Francisco, California

## Abstract

**Objectives:**

In light of the ongoing opioid epidemic, emergency clinicians have faced the difficult challenge of managing acute pain while reducing opioid prescriptions. Improved use of non-opioid analgesics could decrease the need for opioid medications in the management of acute pain. Limited work has been done to systematically produce a comprehensive list of non-opioid pharmaceuticals targeted to specific pain-associated conditions.

**Methods:**

We conducted a scoping review of recent literature for non-opioid pharmaceuticals that could help manage five painful conditions commonly treated in our institution’s ED: abdominal pain; back pain; chest pain; fracture pain; and headache. In November 2023, we identified reviews published from November 2018–2023 in PubMed to curate a list of alternatives to opioids to effectively manage acute pain.

**Results:**

We screened 246 studies that reviewed management approaches for the five chosen conditions that commonly present with pain in the ED and included 23 studies. Acetaminophen and nonsteroidal anti-inflammatory drugs were recommended for all five painful conditions. Ketamine was suggested for abdominal pain, chest pain, and headaches. For back pain, anti-depressants and muscle relaxants were advised. Benzodiazepines and anti-psychotics were indicated for abdominal pain. Triptans, anti-psychotics, and anti-emetics were suggested for headaches.

**Conclusion:**

This review highlights several non-opioid medications for treating acute pain in the ED. The targeted, comprehensive list generated in this study can serve as a practical resource to support alternative-to-opioid programs in EDs by guiding the creation of pharmaceutical order sets tailored to common ED presentations. Ultimately, this tool may help reduce unnecessary opioid exposure and improve patient outcomes in emergency care settings.

## INTRODUCTION

Pain is the most common chief complaint in patients presenting to the emergency department (ED), accounting for 50–80% of all ED visits.^1^–^3^ Given the prevalence of pain and the worsening opioid epidemic in the United States, the approach to pain management in the ED has undergone significant evolution.^4^,^5^ Opioid prescriptions by emergency physicians declined dramatically between 2012–2018 and continued to decrease during the COVID-19 pandemic.^4^,^5^ Many interventions have been developed to alter approaches to pain management among emergency physicians.^6^,^7^ These interventions have largely been successful and correspond to national trends of decreased opioid prescriptions in EDs.^6^–^8^

Despite this progress, further efforts are needed to optimize pain management approaches for patients receiving care in EDs. Patient-centered outcomes such as pain control must be prioritized while reducing the overexposure to opioids.^9^ A growing body of evidence has indicated that non-opioid pharmacological therapies can be used in place of opioids or as an adjunct along with lower doses of opioids to effectively manage pain and reduce the risk of iatrogenic harm to the patient.^10^–^12^ Emergency clinicians can help address the opioid epidemic by using non-opioid pharmaceuticals and other alternatives in the management of acute pain.

Multiple alternatives-to-opioid programs have shown substantial reductions in opioid exposure, hospital admissions, and ED recidivism, with no observed decline in analgesic effectiveness.^13^–^18^ Despite these successes, scalable and readily implementable resources for broad dissemination remain limited. Very few reviews and guidelines have systematically assembled condition-specific tables of non-opioid pharmacologic options.^19^,^20^ We aimed to create a list of non-opioid pharmaceuticals targeting the top five most common pain-related chief complaints in our ED. Patient data from the year prior to the project’s start date revealed the top five pain-related chief complaints at our institution: abdominal pain; back pain; chest pain; fracture pain; and headache. In this review, we detail our methodology in creating a list of non-opioid alternatives to treat five common painful conditions that present to our institution’s ED.

We set out to perform a scoping literature review to identify non-opioid pharmaceuticals to treat five common painful conditions presenting to the ED.

## METHODS

We identified the five most common pain-related chief complaints using patient data at our institution’s ED from January–December 2022: abdominal pain; back pain; chest pain; fracture pain; and headache. Our institution operates the region’s highest volume ED, with over 73,000 patient visits annually. It also serves as the only Level I trauma center for the city and county, providing comprehensive emergency care to a diverse urban population.

### Search Strategy

We developed a search strategy in collaboration with a clinical librarian that combined several main concepts: the pain condition in question; management of that condition; and emergency context (including search terms reflecting acute care management). We conducted our search within the biomedical literature in PubMed. Given our attempts to combine the most up-to-date data regarding management of painful conditions and perform a review that reflects contemporary prescription practices, we limited the search to papers published in the previous five years (November 2018–November 2023). We included narrative review papers that synthesized available evidence on pharmacologic management of acute pain in the ED, with the goal of highlighting current administration practices rather than conducting an exhaustive evidence appraisal. We did not include systematic reviews and primary studies because our aim was to provide practical, high-level pharmacologic pain management approaches for emergency clinicians rather than evaluate pooled evidence or individual trials.

**Population Health Research Capsule**

What do we already know about this issue?

*Non-opioid analgesics can be used for pain management in emergency settings, yet no comprehensive, condition-specific list has been systematically generated*.

What was the research question?

*Which non-opioid medications can be used to manage five common emergency pain complaints based on literature review?*

What was the major finding of the study?

*Twenty-three of 246 studies met criteria, identifying multiple non-opioid options for common emergency pain complaints*.

How does this improve population health?

*This review provides a practical framework that other institutions can use to help reduce unnecessary opioid use for common pain complaints*.

We identified four search strategies that ranged from broad to narrow search criteria. We conducted four searches for each pain condition and decided on a search criterion when the search yielded an appropriate number of papers to create an optimal list of pharmaceutical options across all conditions. Appendix 1 lists the specific search strategies for each iteration and their respective number of results. Once we identified a standardized search strategy across pain conditions, we conducted a search using Boolean operators that combined keywords and Medical Subject Headings terms in Pubmed on November 13, 2023. The results of this search for each pain condition are shown in Appendix 2. We followed the Preferred Reporting Items for Systematic Reviews and Meta-Analyses (PRISMA) guidelines for literature reviews. This review was not registered with PRISMA.

### Exclusion Criteria

Papers were reviewed and excluded at two levels using criteria developed by all authors. At the first level, one author (KL) reviewed titles and abstracts and excluded studies if they did not mention pharmaceutical approaches for pain management; described procedural or regional approaches for pain management; did not describe the pain condition as being within the scope of practice for the emergency clinician (eg, a study that described management of back pain only related to spinal schwannoma); discussed only treatment for a pediatric population; or the study had not been conducted in an acute care/emergency care setting in the United States. We also excluded primary studies and systematic reviews to prioritize narrative summaries that synthesize medication administration approaches in ED pain management. At the second screening level, two authors (AS and SG) reviewed the full text of papers and excluded those that did not include a discussion of specific pharmaceutical interventions to address the painful condition. All included studies with succinct descriptions of their objectives are listed in Appendix 3.

### Data Extraction

Once the articles were identified, two members of the team (AS and SG) reviewed each article to identify non-opioid pharmaceutical approaches that could be used for acute pain management for the specified condition or region of pain. From the literature review, the team developed a list of non-opioid pharmaceuticals for each of the five painful conditions of interest (Appendix 3). Asterisks indicate studies that presented pharmacologic pain management approaches for the ED based on appropriate, setting-specific data; only one study did not explicitly reference the ED but addressed acute management. An ED pharmacist cross-referenced this list of non-opioid pharmaceuticals with the hospital’s formulary to create a list of medications for acute pain management (Table 1). Medications appearing in Appendix 3 were not included in Table 1 when they were not available on our hospital formulary.

### Results

**Figure 1:**
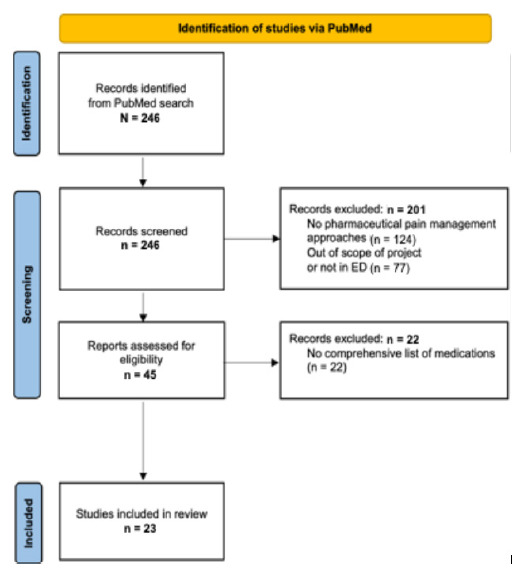
PRISMA flow diagram of scoping review of recent literature for non-opioid pharmaceuticals. *ED*, emergency department. *PRISMA*, Preferred Reporting Items for Systematic Reviews and Meta-Analyses.

A total of 246 studies matched our search criteria for the following conditions that commonly present with pain in the ED: abdominal pain; back pain; chest pain; fracture pain; and headache. Of those, 223 met further exclusion criteria. We included 23 studies in this review (Figure 1).

### Study Characteristics

A variety of conditions were discussed in papers on abdominal pain, including obstruction-related abdominal pain, recurrent abdominal pain (two studies), cyclic vomiting syndrome (two studies), cannabinoid hyperemesis syndrome and other cannabis-related disorders (two studies), small bowel obstruction, and acute pancreatitis. The papers that focused on back pain were not specific to a cause for the back pain. Studies regarding chest pain management either focused on acute chest syndrome secondary to sickle cell disease or pericarditis (two studies). Studies on pain management approaches for headaches either focused on approaches for undiagnosed headaches or migraines. Only one appropriate study was identified for fracture pain, and this specifically focused on pain management for hip fractures in elderly patients with comorbidities.

### Non-Opioid Pharmaceuticals

Table 1 presents the list of non-opioid pharmaceuticals, along with their route of administration and suggested dosage identified through this review.

Non-steroidal anti-inflammatory drugs (NSAID) and acetaminophen were indicated for each of the five painful conditions of abdominal pain, back pain, chest pain, fracture pain, and headache. Antidepressants and muscle relaxants, such as venlafaxine and cyclobenzaprine, respectively, were indicated for back pain. Pharmaceutical pain management approaches for abdominal pain included benzodiazepines, antipsychotics (droperidol and haloperidol), and ketamine. The approach for chest pain also included ketamine. A variety of classes of substances were also indicated for headaches, including antipsychotics (chlorpromazine, droperidol, haloperidol), anti-emetics (metoclopramide, prochlorperazine, promethazine), electrolytes (magnesium sulfate), triptans (sumatriptan), and dissociative anesthetics (ketamine).

## DISCUSSION

In this methodical literature review of non-opioid pharmaceuticals for the management of five painful conditions commonly presenting to EDs, we identified 23 relevant studies that included pharmaceutical pain management strategies. We found some commonalities such as NSAIDs and acetaminophen being indicated for all five conditions, while other medication classes were only indicated for specific conditions (eg, antidepressants and muscle relaxants for back pain). We also found evidence for a diverse set of non-opioid pharmaceuticals, including ketamine, benzodiazepines, and antiemetics, that could be used in the treatment of conditions presenting with abdominal pain and headache.

Using available systematic reviews published after 2019, we created a brief synthesis of the strength of evidence for each medication class by condition in Table 2. We identified several medications with supportive data, including the acetaminophen-ibuprofen combination for all pain-related conditions and select psychotropic agents for migraines. Many other medications had only mixed or preliminary evidence. Still, these findings should not preclude their use. Mixed evidence often reflects heterogeneity in study design or indication for use, and preliminary trials may signal meaningful therapeutic promise.

Other existing alternative-to-opioid pharmaceutical lists provide clear guidance but often lack indication-specific recommendations or a comprehensive review of pharmaceutical options. A broad overview of non-opioid pharmaceuticals substantially overlapped with our list but provided only limited discussion of indication-specific use.^19^ The review discussed lidocaine and gabapentinoids for acute neuropathic pain, while our list uniquely incorporated muscle relaxants and serotonin-norepinephrine reuptake inhibitors. Another set of guidelines offered strong indication-specific recommendations.^20^ For headaches, for example, they also included dexamethasone, benzodiazepines, and muscle relaxants, although they did not address ketamine or antihistamine from our list. For abdominal pain, they added dicyclomine and antihistamines. Recommendations for back pain were largely aligned but, notably, these guidelines did not address fractures or chest pain related to rib fractures—both of which are common ED presentations. These comparisons highlight that, while existing resources offer valuable starting points, our synthesis provides a more comprehensive framework tailored to common ED presentations that can easily be implemented in pharmaceutical order sets.

The list generated from this review addresses pain management approaches for the five most commonly presenting painful conditions at our institution using the hospital’s formulary. It may not address certain pain-associated conditions that are prevalent at other institutions. For example, other pain-associated conditions that commonly present in the national ED data but are not represented in this study include contusions, muscle sprains, other musculoskeletal pain that is not back pain, and urinary tract infections.^64^ In addition, although Appendix 3 compiles all non-opioid pain management alternatives referenced in the literature, Table 1 is limited to medications available within our hospital formulary. Future reviews could provide more comprehensive, indication-specific lists of non-opioid options that are applicable across diverse hospital formularies for other commonly presenting pain-associated conditions.

When generating our list, we chose not to identify the recommended sequence of use for each medication. Some studies included a recommended sequence, but we chose to ignore this information in favor of a general list of treatment options that could be adapted by individual clinicians. Any ED hoping to adopt structural changes, or any individual clinician interested in using more non-opioid medications, can easily integrate this list into practice. An ED could integrate this list into electronic health record tools, and clinicians could use their own clinical judgment on the most appropriate pain management approach for their patient based on the nuances of the patient’s context and presentation.

Along with non-opioid medications, emergency clinicians should also consider the potential benefits of complementary and integrative therapies for pain management in the emergency setting. For example, in the treatment of low back pain, studies also mentioned superficial heat, massage, acupuncture, spinal manipulation, tai chi, yoga, and cognitive behavioral therapy, among other therapies as alternatives to opioids. However, some consideration should be placed on the affordability and practicality of these approaches; these are not available in most EDs, and patients may not have the time, insurance coverage, or funds to pursue these therapies as outpatients.^65^,^66^ Nevertheless, alternative non-pharmaceutical therapies could offer additional options for pain management with potentially lower risks of iatrogenic harm. Future studies should explore the integration of these complementary therapies along with pharmaceutical interventions to provide comprehensive pain management strategies in the ED.

Our findings hold significant implications for pain management approaches in the ED and public health efforts aiming to address the opioid epidemic. By providing alternatives to opioid prescriptions, this review supports efforts to reduce opioid overexposure in the ED while helping to manage patient pain. Implementing these non-opioid pharmaceutical options could potentially mitigate the risks associated with opioid use, including addiction, overdose, and iatrogenic harm, particularly in populations vulnerable to opioid-related adverse events.^67^,^68^ Encouraging the use of these alternatives aligns with broader initiatives aimed at optimizing pain management practices, improving patient outcomes, and contributing to the overall mitigation of the ED’s contribution to the opioid crisis.

The results from this study can be used as a resource in the ongoing education and awareness initiatives in EDs regarding the availability and efficacy of non-opioid pain management strategies. For example, the list of non-opioid pharmaceuticals could be used to develop electronic health record tools that encourage emergency clinicians to heighten their awareness of non-opioid medications for pain management. These decision-support tools would ideally be specific to the clinical scenario, be appropriately timed to clinician workflow, and allow rapid access to ordering specific alternatives to opioids.

## LIMITATIONS

This study has a few key limitations that merit discussion. The medications we identified generally apply to presentations of pain in the five discussed anatomical regions. However, the set of medications within each category does not necessarily represent the best treatment for patients with specific conditions within the broader pain class, such as a schwannoma that presents similarly in a patient as back pain, or acute coronary syndrome vs other causes of chest pain. While emergency clinicians frequently make decisions about their specific pain management approach before the presenting condition is diagnosed, clinicians should be wary of expecting a high degree of efficacy from a non-opioid alternative for non-specific pain. This study also focuses exclusively on non-opioid pharmacologic approaches to pain management. While regional anesthesia techniques are increasingly being used in the ED as a procedural alternative for non-opioid pain control, we did not address this alternative to treat pain. Our aim was to synthesize a pharmacologic toolkit for ED pain management, and while a dedicated review of regional anesthesia techniques would be valuable, it was beyond the scope of this analysis. Future studies could consider the expanding role of regional anesthesia along with non-opioid pharmaceuticals in acute pain management within the ED setting.

Another key limitation of this review was the scarcity of available literature that met criteria for inclusion in our study for certain common pain-associated conditions, such as fracture pain, back pain, and chest pain. This prevented us from creating a comprehensive list of medications that accurately captured appropriate approaches for the management of painful conditions. In addition, for headaches, the literature primarily focused on commonly occurring diagnoses such as migraines, limiting the diversity of conditions included in the review. While the exclusion criteria involved reviewing titles and abstracts to determine whether studies addressed pharmaceutical pain management in the ED setting, another limitation is that the full content of some included studies was not entirely based in the ED or informed by guidelines from emergency care professional societies. However, all included studies addressed pharmaceutical management of acute conditions presenting with pain as a primary symptom, such as intestinal obstruction, low back pain, and cyclic vomiting syndrome. These studies still address pharmaceutical treatment strategies for acute pain presentations that are highly relevant to ED practice.

This review also did not account for the effect size of pharmaceuticals in managing patient pain when compiling the list of alternatives. The primary aim was to identify viable non-opioid alternatives for emergency settings, without assessing the magnitude of pain reduction compared to opioids. In conditions such as acute back pain, where evidence for non-opioid efficacy in the ED remains limited or controversial, the utility of these pharmacologic approaches should be interpreted conservatively. Future research should explore the effect size of these alternatives to ensure comparable pain management outcomes while tailoring prescriptions to individual patient needs and clinical contexts. Future reviews could also include a broader set of studies, rather than only reviews, to help assess efficacy of non-opioid medications.

## CONCLUSION

We identified several non-opioid medications for the treatment of acute pain in the emergency department, which may succeed in treating pain while reducing patient exposure to opioids. Clinicians and EDs may consider using these pharmaceuticals and facilitating their use in electronic health records.

## Supplementary Information







## Figures and Tables

**Table 1:** Alternative to opioid pharmaceuticals for pain management in the emergency department, sorted by commonly presenting pain-associated conditions in the emergency department.

	Product Size	Usual Dose
**Back pain**		
Acetaminophen	Tablet: 325, 500 mg Premix IV: 500, 1000 mg	500–1000 mg IV/PO Q6H
Venlafaxine	Tablet: 75 mg XR	75 mg PO daily
Cyclobenzaprine	Tablet: 5, 10 mg	5 to 10 mg PO TID PRN
NSAIDs		
Ibuprofen	Tablet: 200 – 800 mg Oral liquid	400–800 mg PO Q8H PRN
Naproxen	Tablet: 250, 500 mg	250–500 mg Q12H PRN
Ketorolac	Injectable	15 mg IV Q6H PRN
**Fracture pain**		
Acetaminophen	Tablet: 325, 500 mg Premix IV: 500, 1000 mg	500–1000 mg IV/PO Q6H
NSAIDs		
Ibuprofen	Tablet: 200 – 800 mg Oral Liquid	400–800 mg PO Q8H PRN
Naproxen	Tablet: 250, 500 mg	250–500 mg Q12H PRN
Ketorolac	Injectable	15 mg IV Q6H PRN
**Abdominal pain**		
Acetaminophen	Tablet: 325, 500 mg Premix IV: 500, 1000 mg	500–1000 mg IV/PO Q6H
Benzodiazepines		
Diazepam	Tablet: 2, 5 mg injectable	2–5 mg IV/PO
Lorazepam	Tablet: 1, 2 mg Injectable	1–2 mg IV/PO/SL
Antipsychotics		
Haloperidol	Tablet: 1, 5 mg Injectable	2–5 mg IV/PO
Droperidol	Injectable	1.25–2.5 mg IV/IM
Ketamine	Injectable	0.15–0.3 mg/kg IV/IM
NSAIDs		
Ibuprofen	Tablet: 200 – 800 mg Oral liquid	400–800 mg PO Q8H PRN
Naproxen	Tablet: 250, 500 mg	250–500 mg Q12H PRN
Ketorolac	Injectable	15 mg IV Q6H PRN
**Chest Pain**		
Acetaminophen	Tablet: 325, 500 mg Premix IV: 500, 1000 mg	500–1000 mg IV/PO Q6H
Ketamine	Injectable	0.15–0.3 mg/kg IV/IM
NSAIDs		
Ibuprofen	Tablet: 200 – 800 mg Oral liquid	400–800 mg PO Q8H PRN
Naproxen	Tablet: 250, 500 mg	250–500 mg Q12H PRN
Ketorolac	Injectable	15 mg IV Q6H PRN
**Headache**		
Acetaminophen	Tablet: 325, 500 mg Premix IV: 500, 1000 mg	500–1000 mg IV/PO Q6H
Antipsychotics		
Haloperidol	Tablet: 1, 5 mg Injectable	2–5 mg IV/PO
Droperidol	Injectable	1.25–2.5 mg IV/IM
Chlorpromazine	Tablet: 10, 25 mg Injectable	10–25 mg PO/IM
Prochlorperazine	Tablet: 10 mg Injectable	10 mg IV/IM/PO
Ketamine	Injectable	0.15–0.3 mg/kg IV/IM
Magnesium sulfate	2 G premix IVPB	2 G IV
Metoclopramide	Tablet: 10 mg Injectable: 10 mg	10 mg IV/PO
NSAIDs		
Ibuprofen	Tablet: 200 – 800 mg Oral liquid	400–800 mg PO Q8H PRN
Naproxen	Tablet: 250, 500 mg	250–500 mg Q12H PRN
Ketorolac	Injectable	15 mg IV Q6H PRN
Promethazine	Tablet: 12.5 mg Suppository: 25 mg	12.5–25 mg PO/PR
Sumatriptan	Tablet: 25 mg Nasal spray: 20 mg Subcutaneous: 6 mg	50–100 mg PO 20 mg intranasal 6 mg subcutaneous

*IM*, intramuscular; *IV*, intravenous; *NSAID*, non-steroidal anti-inflammatory drug; *PO*, oral; *SL*, sublingual; *PR*, per rectum; *PRN*, as needed; *Q6H*, every 6 hours; *Q8H*, every 8 hours; *Q12H*, every 12 hours; *TID*, three times daily; *XR*, extended release. *G*, gram; *IM*, intramuscular; *IV*, intravenous; *IVPB*, intravenous piggyback; *NSAID*, non-steroidal anti-inflammatory drug; *PO*, oral; *SL*, sublingual; *PR*, per rectum; *PRN*, as needed; *Q6H*, every 6 hours; *Q8H*, every 8 hours; *Q12H*, every 12 hours; *TID*, three times daily; *XR*, extended release.

**Table 2:** Evidence synthesis of proposed alternative-to-opioid medications for management of commonly presenting pain-associated conditions in the emergency department.

Back pain	Multiple reviews consistently support NSAIDs, both alone and in combination with agents such as serotonin-norepinephrine reuptake inhibitors (SNRI), muscle relaxants, or acetaminophen.^44^–^47^ However, evidence for acetaminophen^48^,^49^ and muscle relaxants individually^45^,^50^ for management of back pain is mixed, with no consistent benefit across trials. SNRIs such as venlafaxine and duloxetine have only limited data, and early studies show small, clinically insignificant improvements, although their use in patients with chronic neuropathic pain suggest they may help patients with similar acute features.^51^
Fracture pain	Initial trials suggest that ibuprofen and NSAID-acetaminophen combinations provide meaningful benefit, whereas the evidence base for acetaminophen alone is inconsistent.^52^–^56^ However, fractures make up only a small portion of randomized trials on acute ED musculoskeletal pain, underscoring the need for more systematic, fracture-specific evidence.
Abdominal pain	Evidence for treating acute undifferentiated abdominal pain remains limited. Acetaminophen outperformed placebo in small trials and shows early pain reductions comparable to opioids.^49^,^57^ For biliary colic and dysmenorrhea, NSAIDs demonstrate the strongest evidence of benefit.^57^ Preliminary studies suggest that neuroleptics^27^ and ketamine may reduce pain intensity,^58^ while benzodiazepines have little supportive evidence outside of abdominal pain related to withdrawal.^59^
Chest pain	Non-opioid management for undifferentiated chest pain is limited, largely because most research focuses on cardiac etiologies. For rib fractures, acetaminophen provides pain relief comparable to other modalities, although NSAIDs have not been formally studied in randomized trials. Ketamine shows mixed results, offering benefit in some older adults but not consistently across the broader population.^60^
Headache	Acetaminophen, NSAIDs, and metoclopramide are commonly used for migraine pain, although recent analyses has questioned the benefit of acetaminophen and NSAIDs.^61^ Antipsychotics such as haloperidol and droperidol can also reduce migraine-associated pain,^62^ and antihistamines are often added to minimize extrapyramidal side effects.^63^ Magnesium sulfate additionally has preliminary evidence suggestive benefits for migraine pain control.^61^

*ED*, emergency department; *NSAID*, non-steroidal anti-inflammatory drugs.
