# Supplementary material for: Non-Opioid Pharmaceutical Alternatives for Acute Pain Management in the Emergency Department: A Scoping Review
Source: West J Emerg Med. 2026 May 14;27(3):659–68. doi: 10.5811/westjem.47925 (PMC13246177; doi:10.5811/westjem.47925)
Supplement: Supplementary file 1 [file wjem-27-659-s001.docx]

**Appendix 1.** Search Strategy Iterations

| **Condition Causing Pain** | **Iteration** | **Search strategy** | **Number of results** |
| --- | --- | --- | --- |
| Abdominal Pain | Search 1 | (“abdominal pain”[ti] AND management[ti] AND emergency[ti] AND undifferentiated) NOT pediatric | 0 Results |
|  | Search 2 | (“abdominal pain”[ti] AND management[ti] AND emergency[ti]) NOT pediatric | 1 Result |
|  | Search 3 | (“abdominal pain”[tiab] AND management[tiab] AND emergency[tiab]) NOT pediatric | 85 Results |
|  | Search 4 | (“abdominal pain” AND management AND emergency) NOT pediatric | 179 Results |
| Back Pain | Search 1 | (“back pain”[ti] AND management[ti] AND emergency[ti] AND undifferentiated) NOT pediatric | 0 Results |
|  | Search 2 | (“back pain”[ti] AND management[ti] AND emergency[ti]) NOT pediatric | 0 Results |
|  | Search 3 | (“back pain”[tiab] AND management[tiab] AND emergency[tiab]) NOT pediatric | 18 Results |
|  | Search 4 | (“back pain” AND management AND emergency) NOT pediatric | 76 Results |
| Chest Pain | Search 1 | (“chest pain”[ti] AND management[ti] AND emergency[ti] AND undifferentiated) NOT pediatric | 0 Results |
|  | Search 2 | (“chest pain”[ti] AND management[ti] AND emergency[ti]) NOT pediatric | 1 Result |
|  | Search 3 | (“chest pain”[tiab] AND management[tiab] AND emergency[tiab]) NOT pediatric | 44 Results |
|  | Search 4 | (“chest pain” AND management AND emergency) NOT pediatric | 137 Results |
| Fracture Pain | Search 1 | (fracture[ti] AND management[ti] AND emergency[ti] AND undifferentiated) NOT pediatric | 0 Results |
|  | Search 2 | (fracture[ti] AND management[ti] AND emergency[ti]) NOT pediatric | 0 Results |
|  | Search 3 | (fracture[tiab] AND management[tiab] AND emergency[tiab]) NOT pediatric | 57 Results |
|  | Search 4 | (fracture AND management AND emergency) NOT pediatric | 308 Results |
| Headache | Search 1 | (headache[ti] AND management[ti] AND emergency[ti] AND undifferentiated) NOT pediatric | 1 Result |
|  | Search 2 | (headache[ti] AND management[ti] AND emergency[ti]) NOT pediatric | 5 Results |
|  | Search 3 | (headache[tiab] AND management[tiab] AND emergency[tiab]) NOT pediatric | 42 Results |
|  | Search 4 | (headache AND management AND emergency) NOT pediatric | 220 Results |

We performed 4 searches across pain conditions to identify a standardized search strategy with the appropriate number of results for this review. We ultimately selected search iteration 3 as the standardized search strategy.
