# Supplementary material for: Non-Opioid Pharmaceutical Alternatives for Acute Pain Management in the Emergency Department: A Scoping Review
Source: West J Emerg Med. 2026 May 14;27(3):659–68. doi: 10.5811/westjem.47925 (PMC13246177; doi:10.5811/westjem.47925)
Supplement: Supplementary file 2 [file wjem-27-659-s002.docx]

**Appendix 2.** Search strategy details.

| **Date** | **Database searched** | **Condition Causing Pain** | **Search strategy** | **Number of results** |
| --- | --- | --- | --- | --- |
| 11/13/2023 | PubMed  (2019-2024) | Abdominal Pain | (“abdominal pain”[tiab] AND management[tiab] AND emergency[tiab]) NOT pediatric | 85 |
|  |  | Back Pain | (“back pain”[tiab] AND management[tiab] AND emergency[tiab]) NOT pediatric | 18 |
|  |  | Chest Pain | (“chest pain”[tiab] AND management[tiab] AND emergency[tiab]) NOT pediatric | 44 |
|  |  | Fracture | (fracture[tiab] AND management[tiab] AND emergency[tiab]) NOT pediatric | 57 |
|  |  | Headache | (headache[tiab] AND management[tiab] AND emergency[tiab]) NOT pediatric | 42 |

We conducted term harvesting, the identification of keywords and controlled vocabulary used in key articles, followed by an iterative process of testing individual search terms to develop our final search strategy. Boolean logic was applied by combining similar terms with OR and using AND between the two concepts. In early search iterations, the [ti] tag was used to limit results to articles with key terms in the title, while [tiab] expanded the search to include titles and abstracts, improving sensitivity to relevant studies not captured by title-only searches. The database search was conducted in PubMed on November 13, 2023.
