# Supplementary material for: Non-Opioid Pharmaceutical Alternatives for Acute Pain Management in the Emergency Department: A Scoping Review
Source: West J Emerg Med. 2026 May 14;27(3):659–68. doi: 10.5811/westjem.47925 (PMC13246177; doi:10.5811/westjem.47925)
Supplement: Supplementary file 3 [file wjem-27-659-s003.docx]

**Appendix 3.** Included articles and detailed study characteristics by pain-presenting condition.

| Author (year of publication) | Study Objective | List of Non-Opioid Pharmaceuticals Mentioned |
| --- | --- | --- |
| **Abdominal Pain** | | |
| Catena et al., 2019^21^* | Guidance on initial management of intestinal obstruction-related acute abdominal pain | IV crystalloids, anti-emetics |
| Daniels et al., 2020^22^* | Biopsychosocial approach to recurrent abdominal pain management | Antidepressants |
| Frazier et al., 2023^23^* | Biopsychosocial management strategies for cyclic vomiting syndrome | Sumatriptan, ondansetron, NK-1 receptor antagonist, antiemetics (promethazine, prochlorperazine), benzodiazepines (lorazepam), antihistamine (diphenhydramine), neuromodulators, IV fluids, sedatives, fosaprepitant, ketamine, ketorolac |
| Gajendran et al., 2020^24^ | Cannabinoid hyperemesis syndrome management and patient education | IVF, electrolyte corrections, ondansetron, promethazine, IV benzodiazepines, lorazepam, haloperidol, capsaicin, aprepitant, olanzapine, triptans, sumatriptan |
| Herlihy et al., 2019^25^* | Management and research insights for cyclic vomiting syndrome | Lorazepam, dicyclomine, PPI, tramadol, IVF with 5-10% dextrose, ondansetron, diphenhydramine, promethazine, triptans (IN sumatriptan). |
| Long et al., 2019^26^* | Optimization of diagnosis and management for small bowel obstruction in emergency care | Anti-emetics, analgesics, IV fluids |
| Miller et al., 2019^27^* | Neuroleptanalgesia in acute abdominal pain management | Neuroleptic drug (haloperidol or droperidol) |
| Razban et al., 2022^28^* | Therapeutic algorithm for managing cannabis-related disorders in emergency care | Metoclopramide, ondansetron, domperidone, benzodiazepines, haloperidol, droperidol, butyrophenone neuroleptics, capsaicin, paracetamol, NSAIDs, oral THC, dronabinol, nabiximols, nabilone + zolpidem, gabapentin |
| Waller et al., 2018^29^* | Evidence-based overview of acute pancreatitis management for emergency physicians | Acetaminophen, procaine, ketamine |
| **Back Pain** | | |
| Jenkins et al., 2022^30^* | Evidence mapping for low back pain management strategies | Paracetamol, NSAIDS, muscle relaxants, antidepressants |
| Martel et al., 2019^31^* | Overview of non-opioid treatment options for neck and back pain in emergency care | NSAIDs, muscle relaxants, duloxetine |
| **Chest Pain** | | |
| Koehl et al., 2022^32^* | Diagnosis and management of acute chest syndrome in sickle cell disease in the emergency department | Empiric antimicrobial therapy, bronchodilators, acetaminophen, NSAIDs, ketamine, diuretic, transfusion, gabapentinoids, NMDA antagonists, Na blocking agents |
| McGuire et al., 2023^33^* | Evaluation and management of pericarditis and myocarditis in the emergency department | NSAIDs, ASA, ibuprofen, prednisone, colchicine, IVIG, azathioprine, anakinra, empiric antimicrobial therapy, prednisone + cyclosporine or azathioprine, corticosteroids |
| Schwier et al., 2020^34^* | Nursing role in managing acute pericarditis in the emergency department | Colchicine, ASA, ibuprofen, indomethacin, ketorolac, corticosteroids |
| **Fracture Pain** | | |
| Li et al., 2020^35^* | Management overview for hip fractures in elderly patients with comorbidities | Paracetamol |
| **Headache** | | |
| Bilhimer et al., 2020^36^* | Review of ketamine for migraine treatment in the emergency department | Ketamine |
| Filler et al., 2019^37^* | Diagnostic strategies for headaches in the emergency department | NSAIDs, dopamine antagonists, chlorpromazine, metoclopramide, prochlorperazine, triptans, DHE, opioids, steroids |
| Giamberardino et al., 2020^38^* | Emergency department approach to headache: Exclusion of secondary causes and tailored pharmacological treatment | NSAIDS, ketorolac, diclofenac, sumatriptan, chlorpromazine, prochlorperazine, metoclopramide, dexamethasone |
| Jesani et al., 2019^39^* | Evidence-based pharmacotherapy for acute migraine management in the emergency department | Prochlorperazine, metoclopramide, chlorpromazine, haloperidol, sumatriptan, ergotamine, dihydroergotamine, ketorolac, diclofenac, IV ASA, magnesium, VPA, propofol |
| Kazi et al., 2019^40^* | Injectable second-line treatments for migraine in the emergency department | Metoclopramide, prochlorperazine, sumatriptan, dexamethasone, ketorolac, diclofenac, dexketoprofen, acetaminophen, dihydroergotamine, VPA, magnesium, propofol |
| Rashed et al., 2019^41^* | Pharmacological interventions for acute primary headache in the emergency department | Prochlorperazine, promethazine, sumatriptan, metoclopramide, haloperidol, dihydroergotamine, aspirin, naproxen, ketorolac, mag sulfate, VPA, dexamethasone, ketamine, propofol |
| Robblee et al., 2020^42^* | Red flags and management options for headache in emergency and inpatient settings | Prochlorperazine, dexamethasone, diphenhydramine, ketorolac, dihydroergotamine, acetaminophen, divalproex, magnesium sulfate, ondansetron, furosemide, ketamine, levetiracetam, acetazolamide |
| Vekhter et al., 2020^43^* | Behavioral interventions for acute headache and pain management in emergency and inpatient settings | Amitriptyline, nortriptyline |

*Citations that provided recommendations for emergency department care.

*NSAID*, Non-steroidal anti-inflammatory drugs
